# A Tau Class Glutathione-*S*-Transferase is Involved in *Trans*-Resveratrol Transport Out of Grapevine Cells

**DOI:** 10.3389/fpls.2017.01457

**Published:** 2017-08-21

**Authors:** Ascensión Martínez-Márquez, María J. Martínez-Esteso, María T. Vilella-Antón, Susana Sellés-Marchart, Jaime A. Morante-Carriel, Elias Hurtado, Javier Palazon, Roque Bru-Martínez

**Affiliations:** ^1^Plant Proteomics and Functional Genomics Group, Department of Agrochemistry and Biochemistry, Faculty of Science, University of Alicante Alicante, Spain; ^2^Biotechnology and Molecular Biology Group, Quevedo State Technical University Quevedo, Ecuador; ^3^Laboratory of Plant Physiology, Faculty of Pharmacy, University of Barcelona Barcelona, Spain

**Keywords:** cell culture, DIGE proteomics, qRT-PCR, glutathione-*S*-transferase, transformation, transport, resveratrol, *Vitis vinifera*

## Abstract

*Vitis vinifera* cell cultures respond to pathogens and elicitors by synthesizing and extracellularly accumulating stilbenoid phytoalexins. Large amounts of trans-resveratrol (*t*-R) are produced when a cell culture is elicited with methylated cyclodextrins (MBCD), either alone or combined with methyl jasmonate (MeJA). *t*-R transport to the extracellular medium, which represents the apoplastic space, would place this antifungal defense right in the battlefield to efficiently fight against pathogen attack. Yet despite their physiological relevance, these transport pathways are mostly unknown. A broad hypothesis-free DIGE-based proteomic experiment of a temporal series of elicited grapevine cell cultures was performed to explore the expression profiles of *t*-R biosynthetic proteins and other co-expressing proteins potentially involved in such a cell response. A correlation between two tau class glutathione-*S*-transferases (GSTs) with several stilbene synthase and phenylalanine ammonia-lyase isoforms, and with the *t*-R metabolite itself, was found and further assessed by a qRT-PCR gene expression analysis. The best candidate, GSTU-2, was cloned from the cDNA of the MBCD + MeJA-elicited grapevine cells and used for *Agrobacterium*-mediated grapevine cell transformation. The non-elicited lines that overexpressed GSTU-2 displayed an extracellular *t*-R accumulating phenotype, but stabilization of *t*-R required the addition to culture medium of adsorbent compounds, e.g., PVP or β-cyclodextrin. The wild-type cell cultures accumulated no *t*-R, not even in the presence of adsorbents. The transient expression of the GSTU-2-GFP fusion proteins in grapevine cells showed localisation in the plasma membrane, and the immunoprecipitation of HA-tagged GSTU-2 revealed its interaction with HIR, a plasma membrane-bound protein. These findings are consistent with a functional role in transport. This is the first report providing several pieces of experimental evidence for the involvement of a specific tau class GST in *t*-R transport to the extracellular medium.

## Introduction

*Vitis* species defend themselves from fungal infection by accumulating phytoalexins and PR proteins (Derckel et al., 1999). A restricted group of stilbenes, whose carbon backbone derives from the *trans*-resveratrol structure (3,4′,5-trans-trihydroxystilbene, *t*-R) (reviewed in Jeandet et al., 2002), is considered the most important grapevine (*Vitis vinifera*) phytoalexin group (Langcake and Pryce, 1977). Occurrence of *t*-R in the human diet in the form of both fresh grapes and wine (Siemann and Creasy, 1992), and its health-promoting properties (Vang et al., 2011), have resulted in this natural compound being intensely investigated over the last 15 years in both the biomedical and plant science fields.

In contrast, knowledge of the stilbenoid metabolism and trafficking in plants is still quite scarce. *t*-R is synthesized from amino acid phenylalanine via the general phenylpropanoid pathway. It is first formed by stilbene synthase (STS) through the condensation of *p*-coumaroyl-CoA with three units of malonyl-CoA (Langcake and Pryce, 1977). Derivatives of *t*-R may subsequently form through photochemical and enzyme-catalyzed reactions (Waldeck, 1991; Hall and De Luca, 2007; Schmidlin et al., 2008), although several steps still remain uncharacterized.

Synthesis of *t*-R and its non-glycosylated derivatives in grapevine tissues can be induced by biotic and abiotic (physical and chemical) elicitors (reviewed in Bavaresco and Fregoni, 2001; Bavaresco et al., 2009). In grapevine cell cultures, elicitor-induced *t*-R biosynthesis leads to the extracellular accumulation of this compound (Liswidowati et al., 1991; Calderon et al., 1993; Krisa et al., 1999; Tassoni et al., 2005; Belhadj et al., 2008; Ferri et al., 2009; Yue et al., 2011), and is particularly abundant when cells are elicited with methylated cyclodextrins (MBCD), either alone (Morales et al., 1998; Bru et al., 2006; Zamboni et al., 2006) or combined with the phytohormone methyl jasmonate (MeJA) (Lijavetzky et al., 2008; Martínez-Esteso et al., 2009, 2011b; Belchí-Navarro et al., 2012, 2013; Almagro et al., 2014), or its structural analog coronatine (Almagro et al., 2015). Currently, there is no experimental evidence for the pathways that transport *t*-R to the extracellular medium in grapevine cells or any other plant system.

In addition to different types of membrane transporters (multidrug resistance –MDR-, multidrug and toxic compound extrusion –MATE-, or ATP binding cassette –ABC- transporters), multidrug resistance-associated proteins (MRP) are also known to be involved in the process of secondary metabolites transport (Bartholomew et al., 2002; Grotewold, 2004; Yazaki, 2005), including glutathione-*S*-transferases (GSTs) (Marrs et al., 1995).

Plant GSTs are a superfamily of proteins that display thioltransferase, peroxidase or isomerase activities (Dixon et al., 2002; Dixon and Edwards, 2006), which are involved in flavonoid metabolism (Kitamura et al., 2004), signaling (Chen et al., 2007), and in responses to biotic and abiotic stress and plant hormones (Moons, 2005). Although most GSTs are soluble enzymes, microsomal (Zettl et al., 1994) and apoplastic (Flury et al., 1996) localizations have also been reported. Their involvement in the trafficking and accumulation of secondary metabolites has been demonstrated for anthocyanins through biochemical and mutant analyses in maize (Marrs et al., 1995), petunia (Alfenito et al., 1998), *Arabidopsis* (Kitamura et al., 2004; Li et al., 2011; Sun et al., 2012), and in functional genomic experiments in *Vitis* (Conn et al., 2008; Gomez et al., 2011). However, failure to detect anthocyanin-GSH conjugates in plant cells (Zhao and Dixon, 2010) strongly supports the hypothesis that a GST acts as a carrier or “ligandin” of anthocyanins rather than a GSH-conjugating enzyme (Mueller and Walbot, 2001). The co-transport of glucosylated anthocyanidin and free GSH occurs in microsomes of yeast-expressing grapevine ABCC1, and leads to the conclusion that GSH conjugation is not an essential prerequisite for anthocyanin transport (Francisco et al., 2013). In grapevine cells treated with MBCD or MBCD + MeJA, the similarity of the protein abundance profiles (Martínez-Esteso et al., 2011b) and gene expression (Almagro et al., 2015) of STSs and GSTs has been noticed, and their coordinated action in the synthesis and transport of *t*-R, respectively, has been suggested.

According to a classification based on sequence similarity and gene organization, both the GSTs involved in anthocyanin accumulation and those co-expressed with STS are tau class (Edwards et al., 2000; Dixon et al., 2002). However, a phylogenetic analysis performed with other GSTs identified in proteomic studies of grapevine berry skin (Martínez-Esteso et al., 2011a) and flesh (Martínez-Esteso et al., 2013), and in elicited cell cultures (Martínez-Esteso et al., 2011b), and those co-expressed with STS clustered apart, indicates a role of specific GSTs in *t*-R accumulation, which is potentially related with the transport of the metabolite to the extracellular medium.

To explore this possibility, we carried out a broader DIGE-based proteomic co-expression analysis to better select the potential GST candidates involved in *t*-R transport. Candidate expression profiles were confirmed by qPCR. One candidate, GSTU-2 (gi| 359473386| XM-002275302.2), was cloned from the cDNA of the MBCD + MeJA-elicited grapevine cells, and was used for *Agrobacterium*-mediated transformation of grapevine cells under the control of a constitutive promoter. The non-elicited transformed lines displayed an extracellular *t*-R-accumulating phenotype, and thus provided strong evidence for the involvement of a specific tau class GST in *t*-R transport to the extracellular medium for the first time. Confocal microscopy studies of the grapevine cells that transiently express a GSTU-2-GPF fusion protein showed both plasma membrane and tonoplast subcellular localisation. The physiological and biotechnological relevance of the results is discussed.

## Materials and Methods

### Plant Material

*Vitis vinifera* L. cv. Gamay calli were kindly supplied by Drs. J. C. Pech and A. Latché (ENSA, Toulouse, France) in 1989. These cell lines were maintained in both solid and liquid cultures in Gamborg B5 medium as described elsewhere (Bru et al., 2006).

### Elicitor or Adsorbent Compounds Treatments

Treatments were carried out in triplicate as previously described (Bru et al., 2006), with slight modifications. A weighted amount of fresh washed cells was transferred into shaking flasks and suspended in fresh growth medium (4 mL/g of cell FW) supplemented with either elicitors (5 or 50 mM MBCD, 100 μM MeJA, 50 mM MBCD +100 μM MeJA), or adsorbent compounds (0, 1.5 and 3 g/L PVP or β-cyclodextrin (BCD), and was maintained at 25°C in a continuous rotary shaker (110 rpm) under a 16 h light/8 h dark photoperiod. The extra- and intracellular stilbene metabolites were extracted as formerly described (Martínez-Esteso et al., 2011a).

### DIGE Analysis

A DIGE analysis, as described by Martínez-Esteso et al. (2011b), was carried out using the soluble protein extracts from the grapevine cell suspensions treated with elicitors (MBCD, MeJA, MBCD + MeJA) for several incubation times. For each time point and treatment, three independent culture flasks were prepared, which were sampled at once and used for the protein, RNA and stilbene extractions to obtain three biological replicates. Details of protein extraction, protein sample preparation, protein CyDye labeling, two-dimensional electrophoresis, image analysis, protein identification by LC-MS/MS and functional annotation are provided as the Supplementary Material.

### RNA Isolation, cDNA Synthesis and Real-Time Quantitative PCR

Total RNA was isolated as described elsewhere (Morante-Carriel et al., 2014) from 1 g of elicited grapevine cells, and was quantified in a Nanodrop ND-1000 spectrophotometer (Thermo Scientific). Only the RNA samples with a 260/280 ratio between 1.9 and 2.1 were used. Residual genomic DNA was removed by DNase I digestion with RNase-Free DNase I (Thermo Scientific). First-strand cDNA was synthesized from 1 μg of total RNA by a cDNA synthesis kit (RevertAid First Strand cDNA Synthesis Kit, Thermo Scientific) according to the manufacturer’s instructions. SYBR Green Real-time PCR Master mixes (Thermo Scientific) were used for qRT-PCR in a StepOne plus instrument (Life Technologies). Gene-specific primers (Supplementary Table S1) for *Vv*GSTU0 (XM_002265191.2), *Vv*GSTU-1 (XM_003634703.1), and *Vv*GSTU-2 (XM_002275302.2) and *Vv*GSTU-3 (XM_002280496.1) were designed using the OligoAnalyzer 3.1 software (IDT, Integrated DNA Technologies). For each primer pair, reaction efficiency estimates were derived from a standard curve generated from a serial dilution of cDNA pooled from each culture flask of the DIGE experiment. For each gene, expression levels were normalized with respect to the grapevine EF1-alpha (XM_002284964.1) gene, used as a reference control as described elsewhere (Reid et al., 2006; Lijavetzky et al., 2008). The RNA samples employed for the synthesis of the qRT-PCR-analyzed cDNA were isolated from three biological replicates of the control, MBCD, MeJA, and the combined treatment (MBCD + MeJA), at 24 h, as indicated above. The correlation coefficients of the 24-h expression ratios in each treatment normalized to the same expression temporal ratio in the control qRT-PCR data were calculated for validation.

### Cloning of *Vv*GSTU-2 cDNA, Construction of the Binary Vector and *Agrobacterium* Transformation

The *Vv*GSTU-2 coding region was PCR-amplified from the cDNA of the MBCD + MeJA elicited cells (Supplementary Table S2). The amplified DNA fragments were cloned into pGEM-T Easy (Promega) following the manufacturer’s instructions and the inserts were sequenced.

The *Vv*GSTU-2 gene was cloned into the pJCV52 vector (Laboratory of Plant Systems Biology; Ghent University, Belgium) and into pEarleyGate 103 (Earley et al., 2006), which led to C-terminal fusion protein products with HA-tag and GFP, respectively, under the CaMV35S promoter using the Gateway cloning system (see the Supplementary Material).

Binary vector pJCV52-GSTU-2 (**Figure 4A**) and pEarleyGate103-GSTU-2 were transferred to chemically competent *A. tumefaciens* strain C58C1 (pGV2260) (Koncz and Schell, 1986) by standard techniques (Sambrook et al., 1989).

### Transient Expression Assay and the Stable Transformation of Grapevine Cells

*Agrobacterium tumefaciens* harboring construct pEarleyGate103-GSTU-2 was used to transiently transform 10 g of *Vitis* calli following the protocol described by Martínez-Márquez et al. (2015), but with a 72-h co-culture and no selection steps. Images of GFP fluorescence from either cells or protoplasts (prepared as described in García-Florenciano et al., 1992) that transiently expressed the GSTU-2 fusion protein were taken by a confocal microscope (Leica TCS SP2; Leica Microsystems, Wetzlar, Germany). GFP fluorescence was viewed by excitation with a 488-nm Argon laser. Fluorescence emissions were detected with spectral detector sets BP 520–555. Serial optical sections were obtained at 1-μm intervals, and projections of optical sections were accomplished with the Leica confocal software. Brightness and contrast were adjusted by Adobe Photoshop 7.0.

The *A. tumefaciens* cultures that contained the constructs for labeling plasma membrane and cytosol were used for subcellular localisation studies. Both constructs expressed the green fluorescent protein (GFP) as either fusion with plasma membrane aquaporin PIP2A (Nelson et al., 2007) for plasma membrane targeting or without fusion for cytosolic targeting.

The stable transformation experiments were performed using the protocol described by Martínez-Márquez et al. (2015) (see the Supplementary Material).

### Molecular Characterization of the Transformed Grapevine Cell Cultures

Protein extracts and genomic DNA were isolated from the *Vitis* calli as formerly described (Martínez-Márquez et al., 2016). The microsomal protein fraction was stored at -80°C until the *in vitro* cross-linking.

#### PCR analysis

Presence of cassette P35S:GSTU-2 and absence of virB genes in the *Vitis* transgenic calli were assessed by a PCR analysis using genomic DNA as a template. Supplementary Table S3 provides the PCR primers and amplification reactions.

The plasmid DNA used in transformation served as a positive control template, while the genomic DNA from the non-transformed wild-type *Vitis* cells was used as a negative control. The PCR products were analyzed by electrophoresis on 1% agarose gels.

#### Western blotting

The precipitated and air-dried soluble protein extracts were or the microsomal pellet was solubilised in 1x SDS-PAGE sample buffer and denatured at 90°C for 5 min. The protein concentration was determined by an RC DC protein assay (BIO-RAD) (Raghupathi and Diwan, 1994). Proteins (50 μg/lane) were resolved by SDS-PAGE and electro-transferred to the Hybond-P PVDF membranes (GE Healthcare). Membranes were probed at 4°C overnight with rabbit monoclonal anti-HA-Tag antibodies (Sigma) at the 1:1000 dilution, and were incubated at room temperature for 1 h with horseradish peroxidase-conjugated goat anti-rabbit IgG (Pierce) at the 1:10000 dilution. Detection was performed by ECL using the Prime Western Blotting Detection Reagent SuperSignal West Dura system (GE Healthcare, Amersham).

### Analysis of Stilbenoids

#### Silica gel TLC

The ethyl acetate extracts (25% v/v) of culture medium after 12 days of cell suspension growth and the *t*-Resveratrol (*t*-R) standard were submitted to TLC on silica gel plates (Merck) using ethyl acetate/hexane (65:35 v/v) as a mobile phase. They were visualized under a 254-nm UV lamp.

#### HPLC-ESI-MS

Stilbenoid quantitative determination was carried out in an Agilent 1100 series HPLC, equipped with UV–vis and ESI-MS detectors as formerly described (Lijavetzky et al., 2008) using a Poroshell 120 EC-C18 column (4.6 × 100 mm 2.7 microns) (Agilent, Palo Alto, CA, United States) and solvents A (0.05% TFA) and B (0.05% TFA in methanol:acetonitrile 60:40 v/v) at a flow rate of 1 ml/min. The gradient consisted of: 0 min, 22.5% B; 4 min, 35% B; 8 min, 40% B; 14 min, 65% B; 19 min, 65% B; 21 min, 22.5% B; 23 min, 22.5% B. The *t*-R and *t*-Piceid (*t*-Pc) standards were purchased from ChromaDex Inc. (Irvine, CA, United States). Calibration curves were generated to quantify these compounds in the samples obtained from cell cultures.

### Cross-Linking and Immunoprecipitation

The microsomal fraction of grapevine cells was prepared by differential centrifugation at 60,000 × *g* (see the Supplementary Material), and was used for immunoprecipitation after resuspending in RIPA buffer (Alcaraz et al., 1990) and incubating at 4°C for 2 h with gentle shaking. Immunoprecipitations were performed by the ImmunoCruz^TM^ IP/WB Optima F System (sc-45043, Santa-Cruz Biotechnology Inc.) following the manufacturer’s instructions. Membrane fractions (400 μg of total protein) were precleared using Preclearing Matrix F (Santa Cruz Biotechnology; cat. no sc-45057). The antibody-matrix complexes were prepared with an anti-HA-Tag antibody (Sigma) (at a 2-μg ratio of antibody per 100 μg of protein) and IP Matrix F. The precleared samples were incubated with them for 30 min at 4°C with gentle shaking. After centrifugation, the pellets that contained the protein-antibody-matrix complexes were carefully washed with PBS and analyzed by SDS-PAGE. Slices of the gel lanes were processed for protein identification as described in the Supplementary Material for 2DE spots.

Cross-linking was performed as described by Staron et al. (2011) with modifications before processing for immunoprecipitation. For the *in vivo* cross-linking, either the transformed or wild-type grapevine cell suspension was incubated at a final 0.5% (v/v) formaldehyde concentration for 20 min at 24°C in a continuous rotary shaker (110 rpm). For the *in vitro* cross-linking, the microsomal fraction, resuspended in 50 mM HEPES at pH 7.5, 5% glycerol, 10 mM acid ascorbic, 100 mM PMSF and Sigma protease inhibitor, was supplemented with formaldehyde at the 0.5% (v/v) final concentration in the same buffer for 20 min at 24°C with gentle shaking.

### Statistical Analysis

The *t*-R production data were statistically analyzed by a one-way analysis of variance (ANOVA), followed by the Tukey’s test in the SSPS package (SPSS Inc., Chicago, IL, United States). *P*-values < 0.05 or *p*-values < 0.01 were considered statistically significant.

## Results

### Paralogues of GST Co-express with the *t*-R Biosynthetic Proteins and Genes, and Correlate with *t*-R Extracellular Accumulation

**FIGURE 1:**
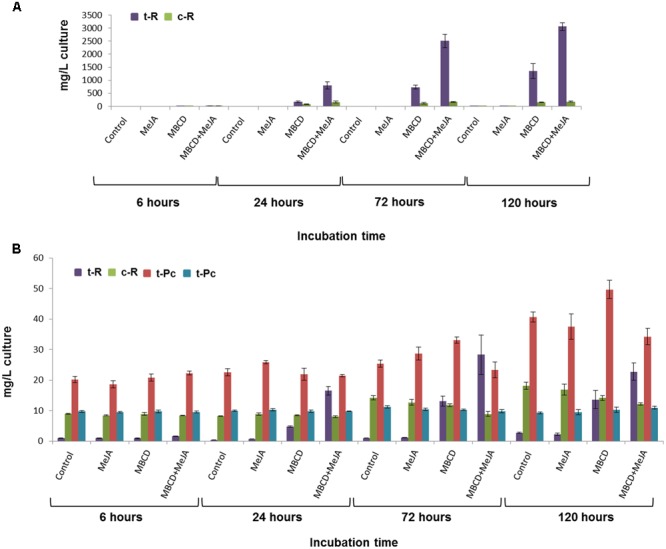
Stilbenoid accumulation by the grapevine cv. Gamay cell culture in the presence of 100 μM MeJA, or 50 mM MBCD, or both elicitors together. **(A)** Effect of the elicitation time course on the extracellular production of trans-resveratrol (t-R) and *cis*-resveratrol (c-R). **(B)** Effect of the elicitation time course on the intracellular production of t-R, c-R, trans-piceid (t-Pc) and cis-piceid (c-Pc). No piceids were detected in the extracellular medium. Data are the mean of four independent replicate experiments ± SD.

A hypothesis-free DIGE-based proteomic experiment of a temporal series of elicited grapevine cell cultures was carried out to broadly explore the expression profiles of the *t*-R biosynthetic proteins and other co-expressing proteins potentially involved in this cell response. Before proceeding to protein extraction, we assessed that the grapevine cell cultures had responded to elicitor treatments, as described in previous studies (Lijavetzky et al., 2008; Martínez-Esteso et al., 2011a). Treatment with MBCD alone or combined with MeJA led to the continuous accumulation of extracellular resveratrol, which reached about 1,500 and 3,000 mg/L of culture, respectively, mainly as the trans- isomer (see **Figure 1A**). In cell extracts, the content of resveratrol was about 100-fold lower, and the glycosylated form of resveratrol, i.e., piceid (Pc), was also detected (see **Figure 1B**). From the four intracellular compounds, only *t*-R followed the trend observed in the extracellular medium, and increased in a time-dependent manner in treatments MBCD and MBCD + MeJA, while the piceid (*cis*- and *trans*-) and c-R content did not respond to treatments. Their concentration did not change compared to the control (e.g., c-Pc), or changed only slightly for longer incubation times (t-Pc increased and c-R decreased). In a previous proteomic study that used DIGE at a fixed 96-h time point, we found that *t*-R accumulation in the MBCD- and MBCD + MeJA-elicited cultures correlated positively with the spots that contained stilbene/resveratrol synthase (STS) isoforms, and also with the spots that contained the tau class glutathione-*S*-transferase (GST) isoforms (Martínez-Esteso et al., 2011b). Since both protein families, STS and GST, contain 10s of members, we decided to carry out a time-series analysis to better distinguish and classify expression patterns.

Four time points, 6, 24, 72, and 120 h, were included consistently with the *t*-R accumulation curve, which leveled off at 96 h and 144 h when elicited with MBCD and MBCD + MeJA, respectively (Lijavetzky et al., 2008). Eventually, the 120-h time point proteomic data were discarded because a pleitropic effect on the differential proteome was observed at this incubation time (Supplementary Figure S1). In all, 212 spots (ANOVA *p* < 0.01; fold change > 1.5) were found with differential abundance across the elicitation conditions and for incubation times up to 72 h; protein identification was successful in 191 spots. Under the *t*-R-producing elicitation conditions (**Figure 1A**), two proteins that catalyzed the first and last committed steps of the *t*-R biosynthetic pathway, respectively PAL and STS (Langcake and Pryce, 1977), were found to be differentially expressed and up-regulated; the former in 6 spots and the latter in 29 spots. Likewise, GST was identified in 20 differential spots, whose abundance profiles followed several patterns (Supplementary Figure S2). All the expression patterns of those spots that contained one single protein were submitted to a hierarchical clustering analysis, except for those spots in which more than one protein type co-migrated.

**FIGURE 2:**
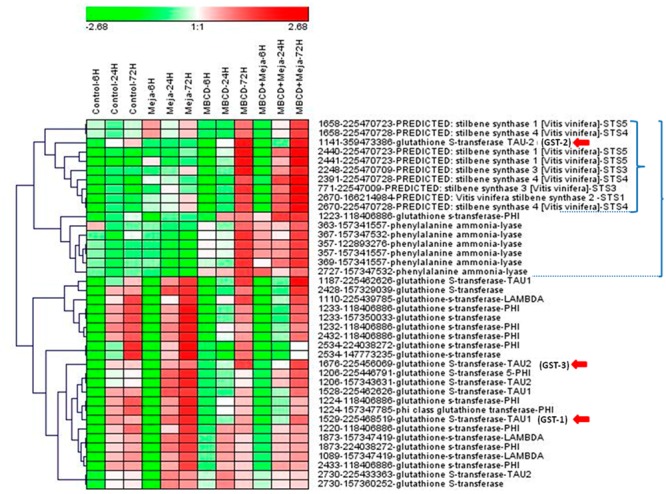
Hierarchical clustering analysis of the protein abundance profiles of the selected spots from a DIGE proteomic experiment. The DIGE experiment is composed of 12 groups, which resulted from combining four elicitation conditions (control, and treatments MeJA, MBCD and MBCD + MeJA) and three sampling times (6, 24, and 72 h). Each experimental group was made up of three biological replicates. Represented values are the log_2_ of the average spot volume of the experimental group in relation to the internal standard across the experiment for such a spot. Spot selection was based on ANOVA *p* < 0.01 and a maximum fold change >1.5. The heat map was created using Genesis v1.0 (Sturn et al., 2002) by clustering spots according to their abundance profile using the average linkage with Euclidean distance. The spot legend includes: spot# - gene index accession number – protein description – protein abbreviation used in this work. A small bracket indicates the cluster formed by mainly stilbene synthases, which contains a glutathione-*S*-transferase (GST). A large bracket indicates the cluster formed by mainly stilbene biosynthesis pathway enzymes, which contains a second GST. Red arrows indicate the GSTs selected for the gene expression analysis (see **Figure 3**).

As shown in **Figure 2**, this set of spots clustered into two major groups: one that contained spots which increased in treatments MBCD and MBCD + MeJA, especially at 72 h, and another that increased in all the treatments in a time-dependent manner, where MeJA was the treatment that brought about the most marked increase. The first cluster included 11 spots that contained STSs and 6 spots that comprised PALs, each grouped into separate subclusters, and two spots that contained GSTs, one of which clustered with STSs and the other with PALs. According to these data, MBCD, but not MeJA, was the major factor for the induction of PAL, STS and two GSTs. Some of the STS isoforms appeared in the spots mildly induced by MeJA.

In the second cluster, 15 GST isoforms were distributed into 17 spots. According to these results, the GST isoform that co-expressed with STSs, which we call here GSTU-2 (gi| 359473386| XM-002275302.2), is a good candidate to be involved in *t*-R production in response to elicitors, and belongs to the tau class (GSTU according to the nomenclature proposed by Edwards et al. (2000). The GSTUs identified in proteomic studies in grapevine separated into two different phylogenetic clusters: members of cluster tau-1 were abundant during veraison in berry skin (Martínez-Esteso et al., 2011a), while members of cluster tau-2 were abundant in the elicited cell cultures (Martínez-Esteso et al., 2011b), including our candidate.

Despite the striking similarity between the GST isoforms, the identification of GSTU-2, and thus of its coding gene, was unambiguously supported by several proteotypic peptides; i.e., peptides not found in any other protein, identified after the MS/MS data from a single spot resolved by 2DE and the database search (Supplementary Figure S3). When BLASTing the GSTU-2 coding sequence against the grapevine genome^1^, a 100% identity with VIT_201s0026g02400 was obtained, which indicates that this locus tag encodes GSTU-2. Next identities of 91% and 92% with the VIT_201s0026g02370 and VIT_201s0026g02390 locus tags was respectively obtained, as was the 88% identity of these two polypeptides at the protein level *vs.* GSTU-2, with 98.5% between them. The three locus tags were located in chromosome 1, in a region of 28 kb. Despite this similarity and genomic clustering, the evidence provided herein revealed that only the GSTU-2 polypeptide accumulated differentially in grapevine cells in response to elicitors MBCD and MBCD + MeJA.

To further assess the correlation found among *t*-R, STS and GST, we performed a real-time qRT-PCR analysis to determine the changes in the transcript profiles of three PAL, five STS and four GST genes after 24 h of elicitation. The PAL and STS paralogues were the encoding proteins identified in the current proteomic experiment. With GST (the red arrows in **Figure 2**), the four tau class paralogues were chosen as follows: one was the candidate tau-2-class (GSTU-2), another was tau-2, but with a different expression profile (GSTU-3), and the remaining two were tau-1; one identified here in cell suspensions (GSTU-1) and in berry skin, and the other identified only in berry skin (GSTU-0) (Martínez-Esteso et al., 2011a). The abundance profile of GSTU-0 during ripening matched both the accumulation of anthocyanins and the abundance profile of its biosynthetic enzyme UDP-glucose:flavonoid 3-*O*-glucosyl transferase.

**FIGURE 3:**
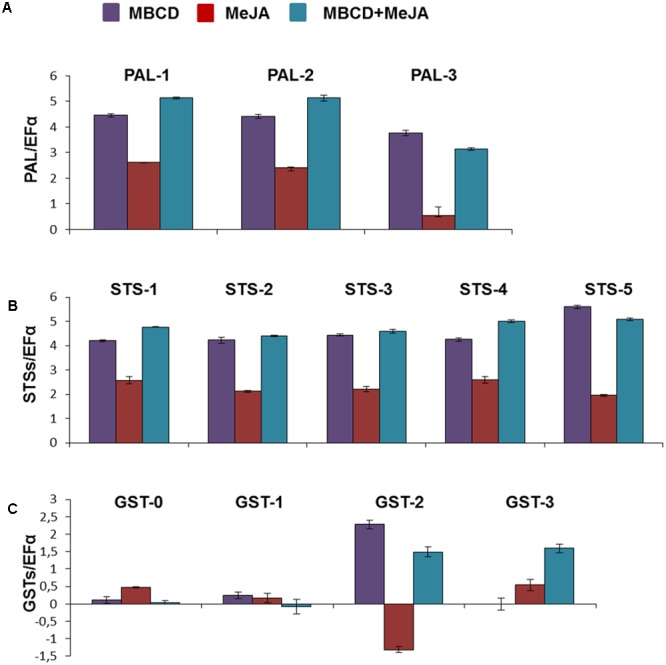
Relative expression of the various PAL **(A)**, STS **(B)**, and GST **(C)** genes in the grapevine cv. Gamay cells in liquid culture elicited with MeJA, MBCD, and MBCD + MeJA at 24 h of treatments. Genes were selected on the basis of the differential abundance in the proteomic experiment in all cases, except for GSTU-0. This was included for comparison purposes as its abundance profile in developing grape berry skin correlated with anthocyanin biosynthesis (Martínez-Esteso et al., 2011b). Transcript levels were calculated using the EF1-alpha gene expression as the internal standard. Represented values are the log_2_ of the elicited/control ratio, given as the mean ± SD of three biological replicates. PAL, phenylalanine ammonia lyase; STS, stilbene synthase; GST, glutathion-S-transferase.

As seen in **Figures 3A,B** genes PAL and STS showed highly similar expression profiles, both qualitatively and quantitatively. The expression of the genes of both groups was greatly enhanced by MBCD or MBCD + MeJA, but less so by MeJA. Such an expression pattern strongly correlated with the production and extracellular accumulation of *t*-R, and with the synthesis of proteins PAL and STS under elicitation by MBCD or MBCD + MeJA, but not by MeJA alone (**Figures 1A,B**). No correlation was found in any case with the abundance of *trans*- and *cis*-piceid (**Figure 1B**) found only within cells, and intracellular *c*-R (**Figure 1B**). The impairment between MeJA gene-induction and *t*-R production has already been observed for two STS genes and one PAL gene (Lijavetzky et al., 2008). One of the four GST analyzed paralogues, the candidate GSTU-2, displayed an expression profile that correlated positively with biosynthetic genes STS and PAL under the *t*-R producing conditions; i.e., MBCD or MBCD + MeJA elicitation. MeJA alone caused the down-regulation of GSTU-2 expression. The GSTU-3 paralogue was not induced by MBCD, only weakly by MeJA, but more strongly by the combination of both, and those of the tau-1 class were practically not affected. Altogether, the co-expression analysis was consistent with the involvement of GSTU-2 in the elicitor-mediated accumulation of large amounts of *t*-R in grapevine cell cultures.

### *V. vinifera cv.* Gamay Cell Cultures Can Be Stably Transformed with GSTU-2 under Constitutive Expressions

**FIGURE 4:**
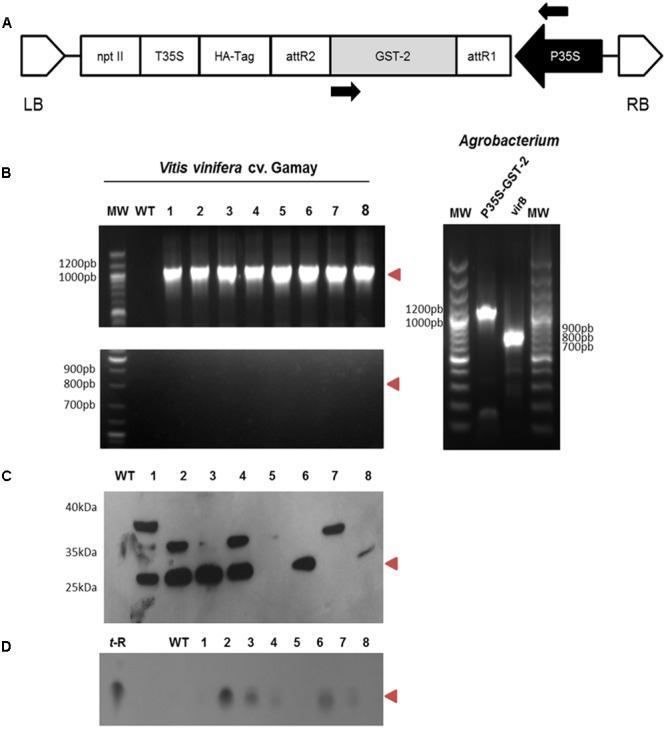
*Agrobacterium*-mediated transformation of the *Vitis vinifera* cv. Gamay cell culture with GSTU-2: genetic construction and molecular characterisation of the transgenic callus lines. **(A)** Schematic diagram of the T-DNA region of the binary plasmid used for the transformation experiments. P35S, CaMV 35S promoter; nptII, neomicine phosphotransferase under the control of the P35S promoter; T35S, CaMV 35S terminator; GST, glutathione-*S*-transferase; LB, left border; RB, right border. **(B)** PCR amplification products of P35S-GSTU spanning the stretch between the arrows in A (top gel), and of virB (bottom gel) from the wild and transgenic lines of *V. vinifera* cv. Gamay using genomic DNA as a template. Positive controls using plasmidic DNA *Agrobacterium* as a template are shown in gel on the left. **(C)** Expression of the GSTU-2 recombinant protein in the cell cultures of *V. vinifera* cv. Gamay. Expression of the HA-tag fusion proteins was confirmed by a Western blot analysis with an anti-HA-tag antibody. **(D)** Extracellular t-R accumulation in the transformed grapevine cell culture in the presence of 1.5 g/L PVP as an adsorbent was confirmed by silica gel TLC. WT, wild-type callus negative control using the non-transformed *Vitis* cells, 1–8 randomly selected transgenic callus lines. Red arrowheads indicate the expected size or migration point.

*Agrobacterium tumefaciens* strain C58C1 (pGV2260) that harbors pJCV52-GSTU-2 (**Figure 4A**) successfully transformed the calli of *V. vinifera* cv. Gamay following the protocols we previously described for other genes of interest (Martínez-Márquez et al., 2015, 2016). After selection on paramomycin the transformed callus lines were established from individual calli. Within 3–4 months of the initial transformation, callus material was used to establish rapidly growing cell suspensions from each transformed line without detectable difference in cell growth compared to the wild parent cell line. The *Vitis* transgenic cultures were maintained under continuous paramomycin selection for more than 7 months, and for 4 months more in the paramomycin-free medium with no loss of vigor. On average, 11 transformed calli were obtained per 1 g FW of plated cells, and no differences were seen between the callus source material. About 60% of the *Vitis* transformed calli were successfully maintained under continuous paramomycin selection.

Eight randomly selected grapevine transgenic calli, as well as the control wild-type callus, were checked for integration of 35S:*Vv*GSTU-2 gene in the plant genome by PCR. They were also tested for *Agrobacterium* contamination using virB primer pairs. GSTU-2 was present in all the transgenic clones, but not in the wild type, whereas the virB PCR product was absent in all the transgenic lines (**Figure 4B**). This scenario proves that the transgenic cultures were transformed with GSTU-2 and were not contaminated by *Agrobacterium*.

Immunodetection of the HA-tagged GSTU-2 protein in the soluble fraction of the corresponding transformed *Vitis* cultures was used to assess recombinant protein synthesis in both transgenic calli and the negative control wild-type callus. The HA-tagged GSTU-2 protein bands were visualized in Western blots with an anti-HA-tag antibody (**Figure 4C**). A HA-tagged GSTU-2 band, with the expected molecular weight of 25.8 kDa, was clearly distinguished in the soluble protein fraction of six of the eight *Vitis* transformant calli, but was absent in the non-transformed cultures. An additional band of approximately 36 kDa was detected in transformant calli 1, 2, 4, and 7, and a band of apparent Mw of 38 kDa was also detected in 1.

Inconsistency among the gene, transcript and protein presence, even under the control of a constitutive promoter such as pCaMV35S, has been previously reported, and this phenomenon has been attributed to fluctuations in environmental conditions and stress, which may affect gene expression, transcript steady-state levels or transgene silencing (Boyko et al., 2010; Kotakis et al., 2010). The immune-reactive bands with Mw were significantly higher than expected, and were also associated with lower *t*-R product levels in subsequent metabolite analyses (see below). We have also reported the occurrence of additional heavier immune-reactive bands in transgenic grapevine cell cultures that constitutively express grapevine gene *Vv*ROMT, but not heterologous human gene *Hs*CYP1B1 (Martínez-Márquez et al., 2016). We attribute this to a modification in homologous transgenic proteins, ROMT, in the previous study, and to GSTU-2 in this case, which probably caused their inactivation. No evidence for modification type has been obtained to date.

### Constitutive GSTU-2 Expression in Grapevine Cells Leads to Extracellular *trans*-Resveratrol Production

*Vitis vinifera* cv. Gamay non-elicited cells are able to constitutively synthesize stilbenoids and store them within cells (Martínez-Esteso et al., 2011b). Our stilbenoid analysis showed that the major storage forms were free *c*-R (10–20 mg/L) and glycosylated *cis*- and *trans*-piceid (30–50 mg/L altogether), with free *t*-R being the least abundant (1–2 mg/L) (**Figure 1B**). Using MBCD as an elicitor, either alone or combined with MeJA, affected only the *t*-R levels, but not its stilbenoid relatives, which led to a continuous increase in *t*-R at the extracellular (**Figure 1A**) and steady-state levels in the intracellular compartments (**Figure 1B**). These results suggest that *t*-R is an intermediate compound that can be either converted into other final products, i.e., *c*-R and piceids, or moved out of cells through competing pathways. Hence when a transport system is present or active, and presumably enhanced by elicitation, the compound would be found outside at high levels. Thus our rationale was that occurrence of extracellular *t*-R under non-elicited conditions could be used as a functional assay for the putative candidate genes and proteins involved in *t*-R transport.

As free *t*-R added to the cell suspension quickly disappeared (Morales et al., 1998), we decided to add adsorbent compounds to the growth medium to stabilize the cell-secreted *t*-R. In a preliminary analysis, the transformed cell lines were used to launch cell suspensions, and were left to grow on standard growth medium supplemented with 1.5 g/L PVP, a soluble polyphenol binding polymer. As a result, *t*-R accumulated in the extracellular medium after 12 days of incubation, as determined by TLC (**Figure 4D**), and displayed an apparent correlation with the GSTU-2 Western blot data (**Figure 4C**). The control wild lines gave negative results. This was the first evidence for the involvement of GSTU-2 in *t*-R transport.

**FIGURE 5:**
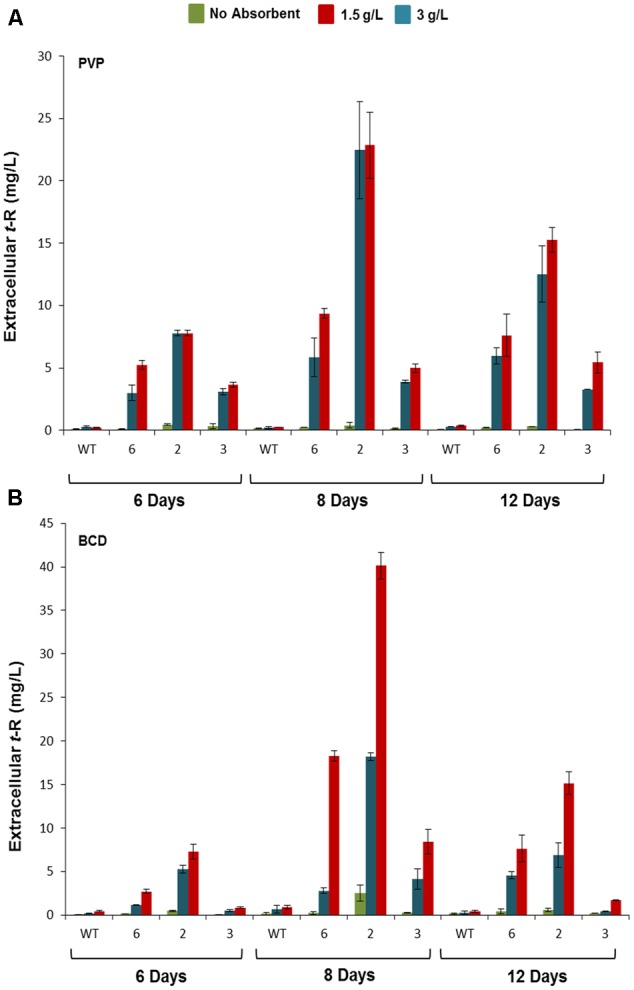
Extracellular accumulation of *t*-R in the p35S:GSTU-2 transformed *Vitis vinifera* cv. Gamay cell culture in the presence of PVP or BCD. Effect of adsorbent compound PVP **(A)** and BCD **(B)** on the extracellular t-R accumulation time course. Accumulation was followed up at 6, 8, and 12 days of treatments in one wild-type and three transformed cell lines (#2, #3, #6). Data are the mean of three independent replicates ± SD.

The three best-producing transformed lines were used to follow up the *t*-R accumulation kinetics by HPLC-UV-MS in the presence of PVP (**Figure 5A**) and other adsorbent compounds, including β-cyclodextrin (BCD) (**Figure 5B**). The latter forms inclusion complexes with *t*-R, as MBCD does, but has no elicitor effect at low concentrations (Bru et al., 2006). Only trace amounts of *t*-R were detected in the extracellular medium of the non-transformed cells in the presence of 1.5 and 3 g /L of either PVP or BCD. In contrast, high extracellular *t*-R levels accumulated in the transgenic lines in the presence of these adsorbents, which increased during the incubation time. This result demonstrates that under non-elicitation conditions, *t*-R transport towards the extracellular medium occurred to a much greater extent in transformed GSTU-2 than in wild-type cells. The highest *t*-R levels were generally found on day 8. The differences in the accumulated *t*-R level at the adsorbent concentrations of 1.5 g/L and 3 g/L were not significant for PVP, but were dose-dependent for BCD. The highest *t*-R production in the transgenic lines treated with PVP and BCD was about 23 mg/L and 40 mg/L, respectively, which represents a 65- and 44-fold increase of the highest production of the wild-type cultures in the presence of the respective adsorbent compounds.

**FIGURE 6:**
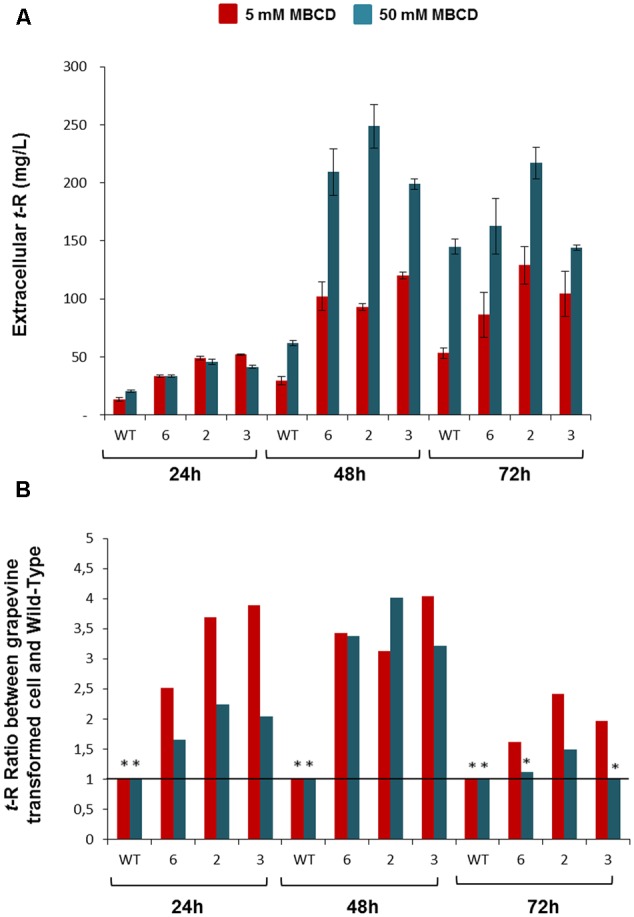
Extracellular *t*-R accumulation in the p35S:GSTU-2 transformed *Vitis vinifera* cv. Gamay cell culture in the presence of elicitor MBCD. The elicitor effect was followed up at 24, 48, and 72 h of treatments in one wild-type and three transformed cell lines (#2, #3, #6) by analysing extracellular t-R production. Two concentrations of MBCD were assayed: 5 mM and 50 mM. Plot **(A)** shows the absolute concentration. Plot **(B)** shows the ratio between the t-R concentration in a transgenic line in relation to the wild type at a given MBCD concentration and incubation time. Data are the mean of three independent replicates ± SD. ANOVA statistical significance below 95 and 90% are indicated as one or two (^∗^).

We also analyzed the elicitor effect of MBCD on the transgenic lines *versus* the wild cells (**Figure 6**). **Figure 6A** shows the amount of extracellular *t*-R that accumulated at 24, 48, and 72 h of incubation with 5 and 50 mM of MBCD. As expected from the elicitor activity of MBCD, abundant extracellular *t*-R was found in both the transgenic and wild-type cell suspensions, but accumulation in the wild type was lower than in the transgenic lines, in which maximum accumulation generally occurred at 48 h. The accumulation level was dependent on the MBCD concentration (Bru et al., 2006). Therefore, in order to better appreciate the difference between the wild and GSTU-2 transformed lines, the values in the wild cultures were taken as the reference to calculate the accumulation ratios in the transformed lines at each incubation time and for each MBCD concentration. As seen in **Figure 6B**, the transformed lines accumulated up to four-fold more *t*-R than the wild cells, with the best effect observed under mild elicitation conditions with 5mM MBCD and at short incubation times up to 48 h, where the constitutive presence of GSTU-2 in the transformed lines was greater than its presence induced by the elicitor effect.

### *Vv*GSTU-2 Is a Membrane-Associated Protein

Transient expression studies with GFP fusions were employed to examine the subcellular localisation of GSTU-2 protein in grapevine cells.

**FIGURE 7:**
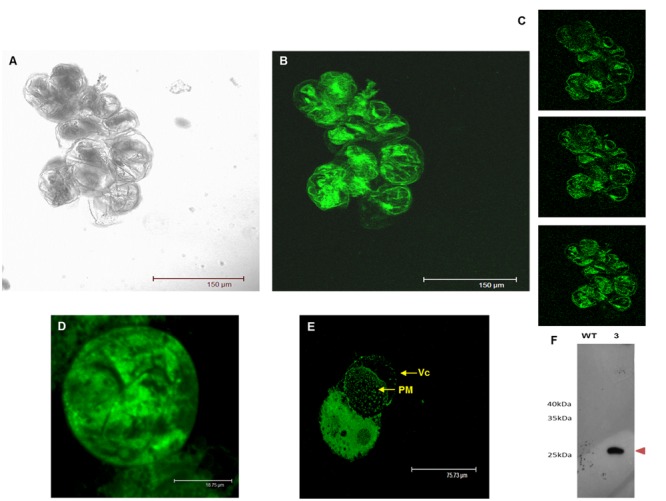
Transient expression of GSTU-2 fused with GFP distributed by grapevine cells and protoplasts 3 days after infection in *A. tumefaciens* of *V. vinifera* cv Gamay. **(A)** Confocal transmission image of the grapevine cell culture. **(B)** Projection of optical sections and **(C)** optical sections showing the intracellular and peripheral localization of the C-terminally GFP-tagged GSTU-2 transiently expressed in the grapevine cell culture. **(D)** Confocal transmission image of the grapevine protoplast prepared from the grapevine cells transiently expressing GFP-tagged GSTU-2. **(E)** Vacuole moving out of a grapevine protoplast through a large fissure in the plasma membrane, leaving behind the fluorescent signal associated with the cytoplasm and the open plasma membrane. **(F)** Expression of the GSTU-2 recombinant protein in the membrane fraction of the cell culture of *V. vinifera* cv. Gamay. Expression of the HA-tag fusion proteins was confirmed by a Western blot analysis with an anti-HA-tag antibody. Vc, vacuole; PM, plasma membrane.

As shown in **Figure 7**, the composite confocal microscopy analysis showed fluorescence distribution in the cell periphery and in internal cell structures (**Figures 7A,B**). To view this distribution better, **Figure 7C** displays three optical slices at different specimen depths, which suggest double localisation in both the plasma membrane and tonoplast. This distribution was compared with the plasma membrane and cytosolic subcellular localisation (Supplementary Figure S4). However, when we observed the large vacuole moved out of the grapevine protoplasts through a large fissure in the plasma membrane (**Figure 7E**), we noticed lack of the fluorescence distribution in the tonoplast, which was left behind, associated with the cytoplasm and the open plasma membrane, thus suggesting a localisation different from tonoplast.

In order to collect further evidence for the interaction of VvGSTU-2 with the plasma membrane, immunodetection and immunoprecipitation experiments with anti-HA-tag antibodies were performed using the membrane fraction of a stable transgenic (line 3), and also the wild-type grapevine cell suspensions that had been subjected to *in vivo* and *in vitro* cross-linking. An HA-tagged GSTU-2 protein band of the expected theoretical mass of 25.8 kDa was visualized in the Western blots with the anti-HA-tag of *Vitis* transformant calli, but was absent in the wild cultures (**Figure 7F**). This provides evidence for the association of VvGSTU-2 with membrane fractions.

Next the mass spectrometry of the protein bands immunoprecipitated after cross-linking and separated by SDS-PAGE (Supplementary Figure S5) led to the identification of a number of grapevine proteins only in the *Vitis* transformant calli, and not in the wild ones (see Supplementary Table S4 for identification details). These include GSTU-2 in a band of 25.8 kDa, along with other proteins in the experiment with *in vivo* cross-linking; e.g.: FBA, Fructose-biphosphate aldolase; GAPDH, Glyceraldehyde 3-phosphate dehydrogenase; VDAC, Porin voltage-dependent anion-selective channel protein; HIR, Hypersensitive-induced response protein; PR10, Pathogenesis-related protein 10 in the experiment of *in vitro* cross-linking and FBA, Fructose-biphosphate aldolase; HIR, Hypersensitive-induced response protein; GLPQ3, Glycerophosphoryl diester phosphodiesterase 3, PER52, Peroxidase 52. The typical contaminant proteins inherent to the experiment, e.g., mouse immunoglobulin G chains, human keratin or porcine trypsin, were also identified in both the transformant and wild materials.

## Discussion

Resveratrol and its derivatives act as antimicrobial compounds, i.e., phytoalexins and phytoanticipins, in grapevine, where they are found in almost every tissue, and accumulate in response to biotic and abiotic stress (reviewed in Jeandet et al., 2002; Bavaresco et al., 2009), and are constitutively present mainly in lignified tissues, but also in soft tissues (reviewed in Bavaresco and Fregoni, 2001). Experiments in grapevine cell cultures have shown that cells synthesize and accumulate *t*-R in the extracellular compartment, which represents the apoplastic space, in response to an array of biotic and abiotic elicitors, including fungal cell walls (Liswidowati et al., 1991; Calderon et al., 1993), MeJA (Krisa et al., 1999; Belhadj et al., 2008; Yue et al., 2011), jasmonates and orthovanadate (Tassoni et al., 2005), chitosan (Ferri et al., 2009), and modified cyclodextrins alone (Morales et al., 1998; Bru et al., 2006; Zamboni et al., 2006) or combined with either MeJA (Lijavetzky et al., 2008; Martínez-Esteso et al., 2009, 2011b; Belchí-Navarro et al., 2012, 2013; Almagro et al., 2014) or coronatine (Almagro et al., 2015). It is therefore reasonable to assume that the site of action of grapevine phytoalexins is the apoplast.

By means of purified compounds or extracellular extracts of elicited cell cultures, it has been shown that stilbenes possess different antifungal properties, including inhibition of mycelia radial growth, inhibition of conidial germination and alteration of morphogenesis in hyphal tips (Hoos and Blaich, 1990; Adrian et al., 1997; Pezet et al., 2004; Bru et al., 2006; Adrian and Jeandet, 2012). This highlights the physiological importance of resveratrol transport for the extracellular compartment to be biologically efficient. Our results showed that the newly synthesized *t*-R was translocated out of cells, and part of the cellular pool of c-R was also probably translocated since the accumulation of small amounts of extracellular *c*-R in the MBCD- and MBCD + MeJA-elicited cells correlated negatively with a significant reduction in the intracellular pool compared with the control and MeJA-treated cells (**Figures 1A,B**). Piceids did not apparently undergo the translocation process as the existing intracellular pools did not change upon elicitation, but continued their growth-linked intracellular accumulation (**Figure 1B**).

Despite their utmost physiological and biotechnological relevance, the *t*-R transport pathways to the extracellular medium in grapevine cells are completely unknown. The present work is thus the first study to aim to find the molecular agents involved in this transport process. The DIGE proteomic analysis at a single time point (Martínez-Esteso et al., 2011b) and at the 24-to-72-h time series, plus a gene expression analysis at 24 h, allowed the discovery of GST as a candidate involved in this process. When we consider that the 2011 release of the annotated grapevine genome contained up to 103 ORFs annotated as GST, which is almost double that in *Arabidopsis* (Dixon et al., 2009), we find it is remarkable that only two isoforms of such a large family fit the profile of *t*-R *de novo* synthesis and extracellular accumulation (**Figure 1**), and the expression profiles of *t*-R biosynthetic enzymes (**Figure 2**).

The role of GSTs in small molecule metabolism and transport has long since been reported in plant systems. GST-catalyzed glutathione (GS) conjugation to small molecules allows the vacuolar sequestration of xenobiotics (Coleman et al., 1997). GST has been demonstrated as an essential factor for anthocyanins to accumulate in vacuoles in the mutants of maize BZ2 (Marrs et al., 1995), petunia AN9 (Alfenito et al., 1998) and *Arabidopsis* TT19 (Kitamura et al., 2004; Li et al., 2011; Sun et al., 2012). However, no potential GS conjugate intermediates have ever been detected (Marrs et al., 1995; Mueller et al., 2000; Zhao and Dixon, 2010). Having demonstrated the flavonoid-binding capacity of GST, a carrier function was proposed for it in an incompletely disclosed transport mechanism of anthocyanins from the cytosol to the vacuole (Mueller et al., 2000; Mueller and Walbot, 2001; Sun et al., 2012). Likewise, we also failed to find [M + H^+^] ions of the *t*-R-GSH conjugates in either the control or elicited grapevine cell cultures. Altogether, these results suggest that some particular GST isoforms could be involved in the *t*-R transport out of cells, in which case possible *t*-R binding rather than GS transferase activity would play a role in transport.

The GSTs known to be involved in transport are classified as multidrug resistance-associated proteins (MRP); i.e., proteins that assist membrane transporters for the extrusion or efflux of metabolites or xenobiotics (Yazaki, 2005). In grapevine, a GST (a putative orthologue of petunia AN9) has been associated with berry color according to co-expression analyses (Ageorges et al., 2006), and was later shown to be involved in anthocyanin transport according to a functional complementation analysis of maize bronze-2 mutant kernels (Conn et al., 2008). An *in vivo* study of anthocyanin transport in grapevine has suggested the involvement of MATE-type transporters, which are closely related with small pigmented vesicles and of the same GST, but not as part of the same transport mechanism (Gomez et al., 2011). Recently, another grapevine GST was shown to rescue the anthocyanin accumulating phenotype in complementation assays of the arabidopsis tt19-1 mutant (Pérez-Díaz et al., 2016). However, these described grapevine GSTs related to anthocyanins are different from GSTU-2. Almagro et al. (2014) described a rise in the transcript levels of transporters *MATE* and *ABC* in elicited grapevine cells, where MeJA and MBCD + MeJA were the treatments to which most of these transcripts responded. In our proteomic analysis, very few membrane proteins, and no MATE-type, were identified because only the cell soluble protein extract was analyzed.

As explained above, occurrence of extracellular *t*-R in transformed grapevine cell cultures under non-elicitation conditions was used as a functional assay for candidate genes and proteins, particularly GSTU-2, which is putatively involved in *t*-R transport. As a result, strong evidence for the involvement of this particular GST in *t*-R transport out of cells was obtained. Incubating cultures with PVP, BCD or MBCD resulted in greater extracellular *t*-R accumulation in transgenic lines than in the wild type (**Figures 5, 6**). While *t*-R accumulation in the presence of PVP or BCD in wild-type cells was negligible compared to transformed cells (**Figures 5A,B**), the wild type significantly accumulated in the presence of MBCD due to the known elicitor effect of MBCD (**Figure 6A**). Nevertheless, markedly enhanced extracellular accumulation was observed in the transformed cultures. This was more pronounced at a lower MBCD concentration (**Figure 6B**), which was expected given the concentration-dependent elicitation strength of MBCD (Bru et al., 2006). The comparison of these experiments also showed that PVP and BCD caused no elicitation at the used concentrations, so the effect was due completely to transgene activity.

The subcellular localization studies carried out herein (**Figure 7**) showed that GSTU-2 interacted with membranes. The immunodetection of the HA-tagged GSTU-2 protein in the soluble fraction, and also in the membrane fraction of the corresponding transformed *Vitis* culture, correlated with and supported microscopy observations. The membrane-associated GSTs also described in plants do not apparently contain membrane targeting or spanning domains, and share a similarity with other GSTs (Zettl et al., 1994). In order to explore the possibility of the post-translational modification of the lipidation type, the GPS-Lipid 1.0 Prediction of Lipid Modification Sites web tool^2^ was used (Xie et al., 2016). The main lipid modifications (*S*-palmitoylation, *N*-myristoylation, *S*-farnesylation, and *S*-Geranylgeranylation) were included in the analysis. However, not even applying the lowest threshold allowed any modifications sites to be predicted. Other membrane docking forms include the interaction with the membrane-bound proteins, as our immunoprecipitation experiments revealed (Supplementary Figure S5, Supplementary Table S4). Interestingly a HIR protein, also known as band 7 protein, was identified in both *in vitro* and *in vivo* cross-linking immunoprecipitation experiments. HIR proteins contain a conserved SPFH domain that appears to assist the formation of microdomains in a variety of cell membranes (Browman et al., 2007). In Arabidopsis, the four known HIR proteins are localized in the plasma membrane, and some are highly enriched in detergent-resistant microdomains and may undergo higher-order oligomerisation (Qi et al., 2011). A role for HIRs as scaffold proteins has been suggested for organizing membrane microdomains and/or recruiting other proteins to membrane microdomains (Qi and Katagiri, 2012). With such a role it is tempting to speculate the hypothesis of an interaction between plasma membrane-bound HIR and GSTU-2 as part of the mechanism of *t*-R transport out of grapevine cells. This interaction is not expected to be permanent since GSTU-2 is also found in the soluble proteome extracted in a detergentless buffer of intermediate ionic strength. These unique reference studies reinforce the proposed role of a specific GST isoform in *t*-R transport, which differ from those involved in anthocyanin transport. The involvement of other elements in the mechanism of *t*-R transport out of grapevine cells, such as membrane transporters and pumps, cannot be ruled out, but future research work is needed.

Besides the present study making progress in knowledge about *t*-R transport, the results might also imply a biotechnological impact if we consider that a major site of action of *t*-R as a phytoalexin is the apoplastic space. Thus engineering the transport system to increase the extracellular *t*-R content in grapevine tissues may have positive consequences for the resistance of economically important grapevine varieties to fungal diseases, which is one of the main worldwide causes of production and quality loss in the grape and wine industry.

From the work presented herein, it can be concluded that GSTU-2 (gi| 359473386| XM-002275302.2) forms part of the not yet discovered machinery for transporting *t*-R to the extracellular medium. Other parts might include membrane microdomains and membrane transporters and pumps, for which some candidates have already been recognized (Almagro et al., 2014) and will be investigated in future work.

## Author Contributions

Conception and design of the work (AM-M, RB-M, and JP); acquisition, analysis, or interpretation of data for the work (AM-M, MM-E, JM-C, MV-A, SS-M, EH, JP, and RB-M); writing of the manuscript draft (AM-M and RB-M); all the authors revised and approved the final version to be published, and agreed to be accountable for all aspects of the work in ensuring that any matter regarding the accuracy or integrity of any part of the work are appropriately investigated and resolved.

## Conflict of Interest Statement

The authors declare that the research was conducted in the absence of any commercial or financial relationships that could be construed as a potential conflict of interest.

## Footnotes

1 http://genomes.cribi.unipd.it/grape/

2 http://lipid.biocuckoo.org
