# Supplementary material for: A Tau Class Glutathione-S-Transferase is Involved in Trans-Resveratrol Transport Out of Grapevine Cells
Source: Front Plant Sci. 2017 Aug 21;8:1457. doi: 10.3389/fpls.2017.01457 (PMC5573539; doi:10.3389/fpls.2017.01457)
Supplement: Supplementary file 1 [file Data_Sheet_1.PDF]

Supporting information:

A tau class glutathione-S-transferase is involved in *trans*-resveratrol transport out of grapevine cells

Martínez- Márquez A. et al.

DIGE analysis

Protein extraction

Protein extracts from *Vitis* cell suspensions were prepared as described (Martínez-Esteso et al. 2011b) with modifications. Briefly, the plant material was homogenized in a potter with teflon pestle in extraction buffer (50mM HEPES, 0.25M sucrose,1% (w/v) PVPP, 5% (w/v) glycerol, 10mM EDTA, 10mM $Na_2O_5S_2$, 10mM ascorbic acid, 1mM PMSF and Sigma Protease inhibitor cocktail) at a ratio of 2mL per gram of plant material at 4 °C. The extract was centrifuged twice, first at 10000×g for 10 min at 4°C and then at 60000×g at 4°C for 90 min, and the supernatant transferred to new tubes. An equal volume of Tris-saturated phenol pH 7.5 (Applichem, Darmstadt, Germany) was added and the mixture incubated for 30 min with vortex every 5 min. Sharp phase separation was achieved by centrifugation at 15700xg for 10 min. The upper phenol phase was recovered and the aqueous phase was submitted to a second phenol extraction. Both phenol phases were pooled and washed twice with an equal volume of phenol washing buffer adjusted to pH 8.0 (0.1M Tris, 20mM KCl and 10mM EDTA). The recovered phenol was precipitated overnight with 5 vols of 0.1M ammonium acetate in methanol. The precipitate was thrice washed with 0.1M ammonium acetate in methanol and twice with 80% acetone (v/v).

Protein sample preparation and labeling of proteins with CyDye

Protein sample preparation was prepared as described (Martínez-Esteso 2011a) 2 g grapevine elicited cells, and quantitatively precipitated according to Bensadoun and Weinstein, 1976. The pellet was washed three times in 80% (v/v) of chilled acetone, left to dry and the precipitated protein was treated according to Deshusses et al. 2003 with some modifications. Briefly, protein pellet was resuspended in 50 mM ammonium bicarbonate and every 150 µl of the resuspended protein was added 1ml of

trifluoroethanol (TFE): chloroform 2:1 (v/v) mixture with strong shaking and was maintained at 4 °C for 1 h with periodical vortexing. Centrifugation at 10,000×g for 5min separated the mixture into three phases. The upper aqueous phase was separated from the insoluble interphase and the lower chloroformic phase. Then, the upper aqueous phases recovered were pooled and concentrated by vacuum centrifugation. Two washes with 80% (v/v) acetone were performed to the dried protein pellet.

Precipitated and air-dried proteins were solubilized in labeling buffer (7 M Urea, 2 M Thiourea, 30mM Tris–HCl, 4% CHAPS, pH 9.0). Insoluble material was pelleted by centrifugation (12,000×g, RT, 5 min) and protein concentration in the supernatant was measured using the RCDC method (Bio-Rad). Two hundred micrograms of protein was adjusted to 1 µg/µl with labeling buffer and further cleaned with Ettan 2D Clean-up kit (GE Healthcare) according to the manufacturer's recommendations. The recovered precipitated protein was solubilized in 30 µl of labeling buffer, the pH was adjusted to 8.5 using NaOH 100 mM and the protein content was determined again as above. For labeling of the proteins, 400 pmol of CyDye in 1µl was mixed with 18 µl of sample containing 50 µg of protein and incubated on ice for 30min in the dark. The labeling reaction was terminated by adding 1 µl of 10mM lysine. Each sample was covalently labeled with a fluorophore either Cy3 or Cy5. A mixture of equal amount of protein from every sample in the experiment was labeled with Cy2 and used as internal standard.

Two-dimensional electrophoresis and DIGE image analysis

Twenty microlitre each of Cy3, Cy5 and Cy2 labeled sample (150 µg of protein) was combined, mixed with 60 µl of sample buffer (7 M Urea, 2M Thiourea, 30 mM Tris–HCl, 100 mM DTT, 1 % IPG buffer (v/v)) and incubated for 10 min on ice. Then, samples were adjusted to 340 µl rehydratation buffer (7 M Urea, 2 M Thiourea, 30 mM Tris–HCl, 2% CHAPS (w/v), 50 mM DTT, 0.5% IPG buffer (v/v), 0.005% bromophenol blue). Samples were then applied to IPG strips (18 cm; pH 4–7; GE Healthcare) for passive

rehydration overnight at room temperature. The rehydrated IPG strips were subjected to isoelectric focusing at 20 °C on an IPGphor Unit (GEHealthcare/Amersham Biosciences) until an accumulated voltage of 50 kVh was achieved. Strips were incubated in reducing equilibration buffer (50mM Tris–HCl, pH 8.8, 6 M Urea, 30% glycerol (v/v), 2% SDS (w/v) and 1% DTT (w/v)) for 30min and subsequently in alkylation equilibration buffer (same as before, but 1% (w/v) dithiothreitol replaced by 1.25% (w/v) iodoacetamide) for 30 min. SDS-PAGE was done as second dimensional separation in 12.5% acrylamide gels in an Ettan Dalt-six (GE Healthcare/ Amersham Biosciences) vertical unit. The separation was run overnight: first step at 80 V, 10 mA/gel and 1 W/gel for 1 h, second step at 150 V, 18 mA/gel and 3 W/gel overnight and third step at 500 V, 38 mA/gel and 13 W/gel until bromophenol blue line reached to the bottom of gel. Images of the Cy3-, Cy2- and Cy5-labeled samples were acquired in a Typhoon 9410 laser scanner (GE Healthcare/ Amersham Biosciences) according to the manufacturer's recommendations. The gel images were analyzed using Progenesis SameSpots v4.0 software (Non-linear Dynamics, Newcastle, UK). First, images were aligned. Briefly, prominent spots were used to manually assign approximately 60 vectors to digitized images within each gel and then automatic vector tool was used to add additional vectors (ca. 600 total vectors) which were manually revised and edited for correction if necessary. These vectors were used to warp and align gel images with a reference image of one internal standard across and within each gel. After automatic spot detection, spots were manually revised with edition tools for a correct detection. Gel groups were established according to the experimental design and spot normalized volume was used to select statistically significant (ANOVA or T-test $p<0.05$) differential spots between treatments applied to grapevine cells. The abundance patterns of the selected spots were analyzed and grouped by hierarchical clustering, assessed by principal component analysis implemented in Progenesis SameSpots.

<u>Protein identification by LC-MS/MS and functional annotation</u>

The protein spots selected through the DIGE analysis were excised manually from the Coomassie-stained gels and subjected to in-gel trypsin digestion (Shevchenko et al., 1996) in a Progest (Genomic Solutions, Cambridgeshire, UK) automatic in-gel protein digestor according to the manufacturer's recommendations for CBB-stained samples. The gel plugs were extensively washed to remove dye and SDS impurities with 25 mM ammonium bicarbonate, in-gel reduced with 60 mM dithiothreitol, and S-alkylated with excess iodoacetamine followed by digestion with porcine trypsin (Promega, Madison WI) (1:100 wt/wt) at 37 °C for 6 h. Peptides were extracted in ammonium bicarbonate, then in 70% acetonitrile, and finally in 1% formic acid. Extracted peptides were dried down in a Speed-Vac benchtop centrifuge and resuspended in 0.1% formic acid (typically 10 µL).

MS and MS/MS data were acquired in an Agilent XCT plus ion trap mass spectrometer with a ChipCube interface fed by an Agilent 1100 series nanopump HPLC system. The sample was concentrated, desalted, and resolved using the ProtID-Chip-150 (II) (Agilent) as described Martinez-Esteso et al., 2011a. MS spectra were scanned at 26,000m/z per second and MS/MS spectra at 8100m/z per second in the range 300–2200 m/z. The 4 most intense precursor ions in MS scans were selected for MS/MS and then passed to an active exclusion list released after 1min.

Each MS/MS spectra data set (ca. 1200 spectra/run) was processed to determine monoisotopic masses and charge states, to merge MS/MS spectra with the same precursor ($\Delta m/z<1.4$ Da and chromatographic $\Delta t<15$ s) and to select high quality spectra with the Extraction tool of SpectrumMill Proteomics Workbench (Agilent). The reduced data set was searched against the NCBInr forward and reversed protein database without taxonomical restrictions in the identity mode with the MS/MS Search tool of SpectrumMill Proteomics Workbench using the following parameters: trypsin, up to 2 missed cleavages, fixed modification carbamidomethylation of cystein, variable modificacion oxidation of methionine, and a mass tolerance of 2.5 Da for the precursor

and 0.7 Da for product ions. Peptide hits were validated first in the peptide mode and then in the protein mode according to the score settings recommended by the manufacturer.

As protein sequences released from whole genome sequencing of grapevine projects (Jaillon et al. 2007; Velasco et al. 2007) were still unnamed at the time of analysis, the positive matches to that type of sequences were submitted to description retrieval and annotation using Blast2GO v2.3.6 (Götz et al., 2008) as described in previous studies (Martinez-Esteso et al. 2011b).

Construction of the binary vector

Briefly, pGEM®-T Easy plasmid harboring the target cDNA sequence was used as a template to amplify by PCR using the proofreading Pfx DNA polymerase (Invitrogen) and the appropriate primers for *Vv*GST-2 (upstream 5´-CACCATGGAGAAGCAAAGTG-3´; downstream 5´-AGCTTTAGGTGGCTGCAG-3´). PCR products were inserted into the pENTR™/D-TOPO® vector (Invitrogen) as recommended by the manufacturer. Plasmids pENTR™/D-TOPO® containing cDNA inserted in the correct orientation were selected by PCR and the complete insert was subsequently sequenced. Correct inserts were transferred into the Gateway-compatible vectors pJCV52 and pEarleyGate 103 using an LR clonase reaction (Invitrogen) carried out according to the manufacturer’s instructions. In the binary vector pEarleyGate103-GST-2, the green florescent protein (GFP) C-terminally fused to the *Vv*GST-2 protein was used for subcellular localization in a transient expression assay in grapevine cell culture. The pJCV52-GST-2 was used for stable transformation of grapevine cell cultures.

Stable transformation of grapevine cell culture

50 milliliters of YEB medium (Maniatis et al., 1982) was inoculated with 1mL of this 1-day-old culture expressing GST-2 constructs and grown until OD_{600}~0.1 according to

Batoko et al. (2000). 10g of *Vitis* callus was transferred into a sterile Erlenmeyer flask containing 50 mL of Gamborg B5 medium supplemented with 100 $mg.L^{-1}$ acetosyringone (AS) and sonicated. Immediately, the bacterial suspension was added and the infected culture was incubated for 30 min in the dark at 24ºC on a shaker (110 rpm). Subsequently, callus pieces were poured over a glass filter and the retained biomass was washed with cold medium. A gentle vacuum was applied to remove the excess liquid medium. The biomass was drained for 5 min on sterile paper, then transferred onto solid Gamborg B5 medium containing 100 $mg.L^{-1}$ AS and after two days of co-culture, cells were transferred onto solid Gamborg B5 medium containing 250 $mg.L^{-1}$ cefotaxime and 60 $mg.L^{-1}$ paramomycin. Periodical sub-cultures of growing callus colonies were carried out with cefotaxime at decreasing concentrations.

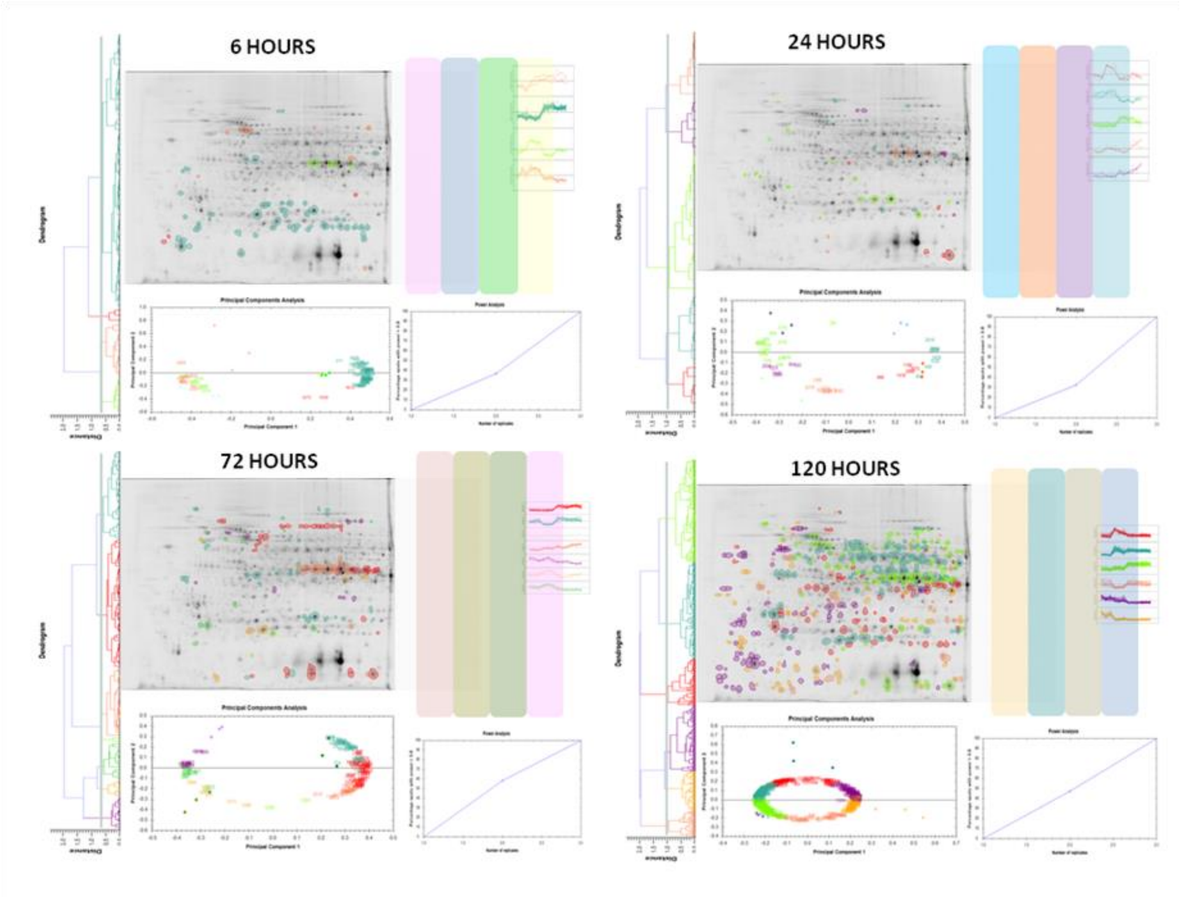

Figure S1: DIGE analysis of elicited grapevine cv. Gamay cells in liquid culture. 2D gel reference image showing the location of the selected spots and pertaining to a cluster by a color code. Dendrogram of spots made by in Progenesis Samespots-implemented hierarchical cluster analysis of abundance profiles across treatments, showing the selection of four clusters. Standardized expression profiles of the spots in four replicates across the control and the treatments MeJA, MBCD and MBCD+MeJA were grouped in four different clusters. PCA bi-plot of the two first principal components. Power analysis.

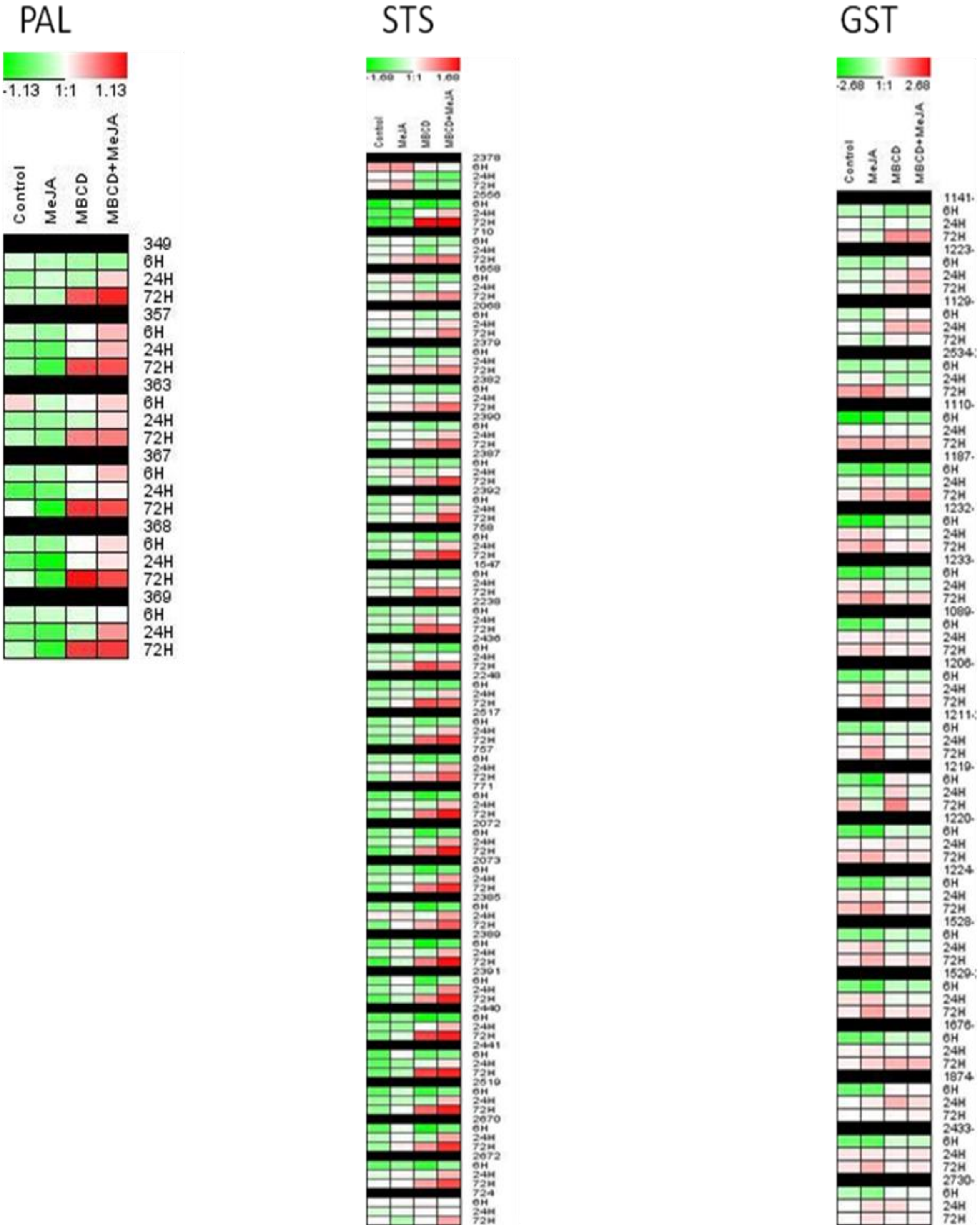

Figure S2: Typical protein of interest and profiles of protein functional clusters of elicited grapevine cv. Gamay cells in liquid culture.

```
CLUSTAL O(1.2.4) multiple sequence alignment

VIT_201s0026g02370.1      MEKQSEVKLFGTWSSGYCTRIKLALKLKGIPYEYVEEDLSNKSDLLIHHNPVHKKVPVLV
VIT_201s0026g02390.1      MEKQSEVKLFGTWSSGYCTRIKLALKLKGIPYEYVEEDLSNKSDLLIHHNPVHKKVPVLV
VIT_201s0026g02400.1      MEKQSEVKLFGTWASVYCTRIELALKLKGIPYEYVEEDLPNKSDLLIHHNPVHKKVPVLV
                          *************:* *****:***************** ********************

VIT_201s0026g02370.1      HNGKAIAESLVILEYIDEHWNHTPKLLPEDPYERAKVRFWANFYDQKFKPSIYNIMTSKG
VIT_201s0026g02390.1      HNGKAIAESLVILEYIDEHWNHTPKLLPEDPYEKAKVRFWANFYDQKFKPSIYNIMTSKG
VIT_201s0026g02400.1      HNGKAIAESLVILEYIDEHWNHTPKLLPADPYERAKVRFWANFYDQKFGPSIYNIMTSKG
                          **************************** ****:************** ***********

VIT_201s0026g02370.1      KEQEKAIEDSREVLMVFEEGIERDFPAKSPFLNGGPLGFLDIVVGSSACNYKAMNEVVGV
VIT_201s0026g02390.1      KEQEKAIEDSREVLKVFEEGRERDFPAKSPFLNGGPLGFLDIVVGSSACNYKAMNEVVGV
VIT_201s0026g02400.1      KEQEKAIEDSLEVLKVFEEGIERDFPAKSPFLNGGTLGFLDIVVGSSSCNFKAFNEVFAV
                          ********** *** ***** ************** ***********:**:**:***..*

VIT_201s0026g02370.1      VVDPKKNPAFCYWMAAMKDCPLMKETLPPHDRLVAKMRSKFSLQPPKT
VIT_201s0026g02390.1      VVDPKKNPAFCYWMTAMKDCPLMKETLPPHDRLVAKMRSKFSLQPPKT
VIT_201s0026g02400.1      VFDPEKNPAFCSWVTAMKECPLMKETLPPHDRLVAKISKLF-LQPPKA
                          *.**:****** *::***:*****************:  . * *****:
```

Figure S3: Alignment of peptide sequences encoded by three grapevine genome locus tags. The locus tag in bold encodes GSTU-2, and the two other are the most similar ones. In red, tryptic proteotypic peptides of GSTU-2 identified in the DIGE proteomic experiment.

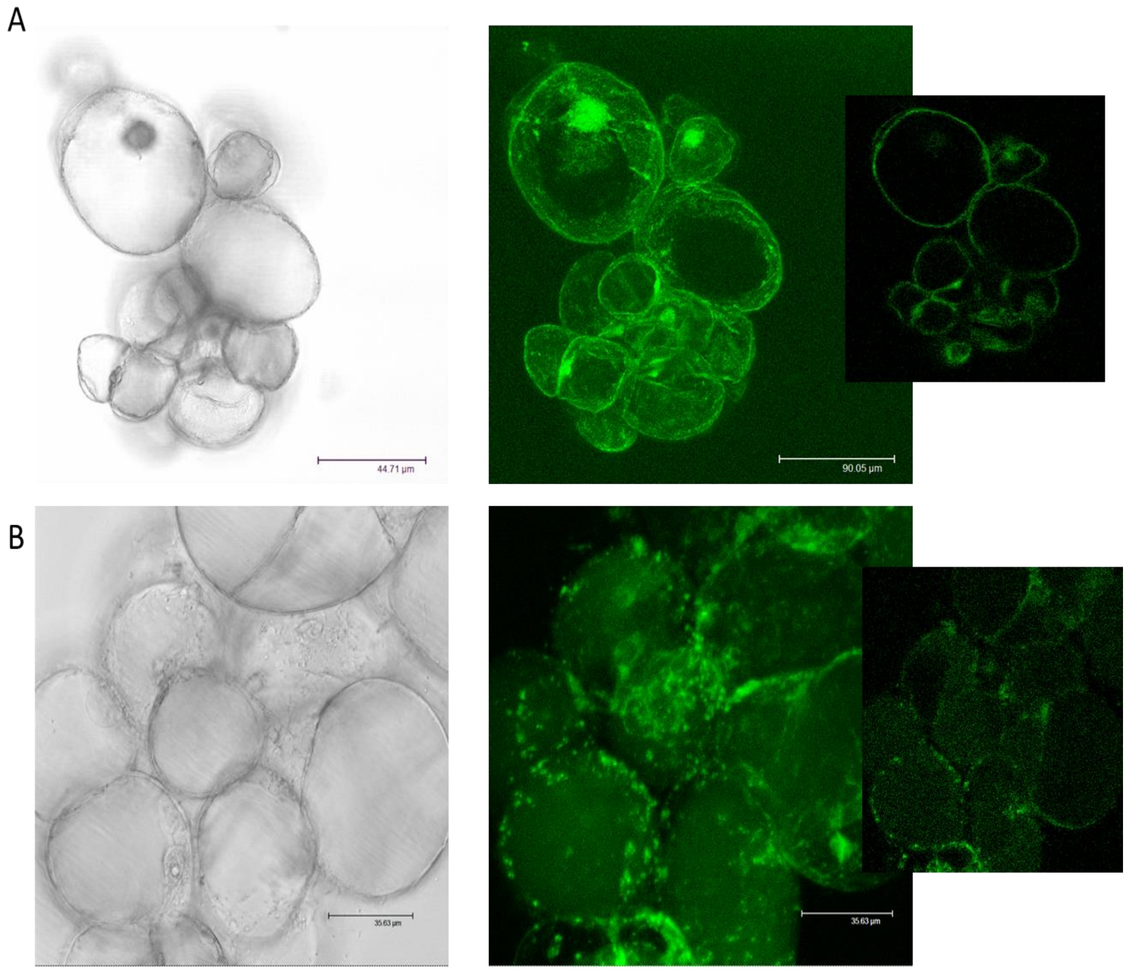

Figure S4: Transient expression of GFP distributed by plasma membrane (A) and cytosol (B). Confocal transmission image of grapevine cell culture, projection of optical sections and optical sections

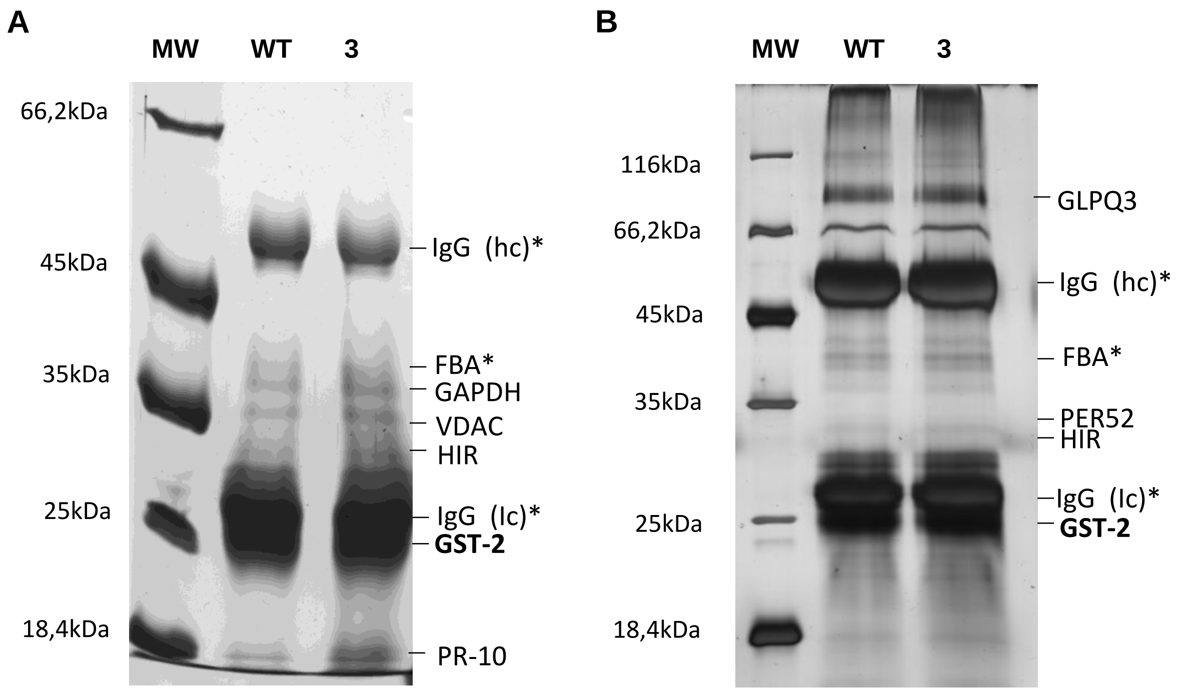

Figure S5: Co-purification of *Vitis vinifera* cv. Gamay GST-2 protein associated with other proteins of membrane fraction. SDS-PAGE gel containing membrane fraction purification with anti-HA-tag antibody (cross-linking *in vitro* and *in vivo*, respectively). Proteins identified by mass spectrometry are indicated. WT, wild-type callus negative control using non-transformed *Vitis* cells; 3, transgenic callus lines. FBA, Fructose-biphosphate aldolase; GAPDH, Glyceraldehyde 3-phosphate dehydrogenase; VDAC, Porin voltaje-dependent anion-selective cannel protein; HIR, Hypersensitive-induced response protein; PR10, Pathogenesis-related protein 10; GLPQ3, Glycerophosphoryl diester phosphodiesterase 3; PER52, Peroxidase 52. Red arrowheads indicate the expected size or migration point. * Protein identified in transgenic and wild-type callus.

Table S1: PCR primers used to amplify gene-specific regions for expression analyses.

	Forward	Reverse	pb
GST0	5'- CCAGTTCTGATCCACAATGGAAAG-3'	5'- CTTCTTGTCTATGTAGTCCGCCC-3'	153
GST1	5'- GCAGGAAACAGCCAAGAAGG-3'	5'- CACCAGAGCCACATCTACAAACC-3'	115
GST-2	5'-GGAGGCACCTTGGGATTTCTC -3'	5' –GCATTCTTTCATAGCGGTCACCC-3'	140
GST-3	5'-GAAGGCTATGGAGTCAGGACTAG-3'	5'-CAACCCACTACCAGATCCAAGA-3'	111
EFα1	5´-GAACTGGGTGCTTGATAGGC-3´	5´-AACCAAAATATCCGGAGTAAAAGA-3´	

Table S2: PCR primers used to amplify *Vv*GST-2 gene from cDNA of MBCD+MeJA elicited cells. The amplification reactions consisted of 1 cycle at 94ºC for 2 min and 30 cycles at 94ºC for 30 s, 55ºC for 30 s, 72ºC for 1 min, followed by an extension cycle of 10 min at 72ºC.

	Forward	Reverse
*Vv*GST-2	5´-ATGGAGAAGCAAAGTGAAGTGAAG-3´	5´-TTTCTGCAGCCACCTAAAGCTTGA-3'

Table S3: PCR primers used to amplify P35S:GST-2 (fragment of the cassette spanning the promoter and the coding region) and virB genes. The amplification reactions consisted of 1 cycle at 95ºC for 5 min and 30 cycles at 94ºC for 1min, 54ºC for 1min, and 72ºC for 1:30 min, followed by an extension cycle of 10 min at 72ºC.

	Forward	Reverse	pb
P35S:GST-2	5´-GCACCTACAAATGCCATCA-3´	5´-AGCTTTAGGTGGCTGCAG-3´	1074
virB	5´-TCGGGCACCGTCAGCTTGACG-3´	5´-GTTAAGAAGATCGCCTATTGT-3´	800

Table S4: Proteins identified after cross-linking in membrane fraction of grapevine cells. *In vitro*-White/ *In vivo*- Blue

Spectra number	Peptides number	Distinct Summed MS/MS Search Score	% AA Coverage	Total Protein Spectral Intensity	Accession number	Protein description
4	3	47.15	13.1	6.19e+007	XP_002283381.1	Fructose-bisphosphate aldolase
2	2	37.35	9.4	2.92e+007	XP_002263145.2	Glyceraldehyde 3-phosphate dehydrogenase
3	3	36.43	13.4	9.28e+007	XP_002272267.1	Hypersensitive-induced response protein 1 isoform X1
2	2	30.86	9.6	5.58e+008	XM_002275302	Vitis vinifera glutathione S-transferase U10-like
3	2	27.44	15.8	2.60e+008	XP_002274242.1	Pathogenesis-related protein 10
3	3	33.50	6.3	1.21e+007	XP_002276757.1	Glycerophosphoryl diester phosphodiesterase GDPDL4 isoform X1
4	3	52.67	15.3	1.02e+008	XP_002283381.1	Fructose-bisphosphate aldolase
5	4	50.05	20.2	1.69e+008	XP_002269918.1	peroxidase 4
5	5	75.52	19.9	1.76e+008	XP_002272267.1	Hypersensitive-induced response protein 1 isoform X1
7	7	100.82	28.6	2.17e+009	XM_002275302	Vitis vinifera glutathione S-transferase U10-like
